# Benchmarking of signaling networks generated by large language models

**DOI:** 10.1101/2025.07.28.667217

**Published:** 2025-07-29

**Authors:** Jeevan Tewari, Benjamin W. Dahl, Jeffrey J. Saucerman

**Affiliations:** 1 co-equal contributors; 2 Department of Biomedical Engineering, University of Virginia

## Abstract

Computational models of signaling networks provide frameworks for predicting how molecular cues guide cell decisions. But they are typically limited by manual curation from incomplete literature. Here, we test whether general-purpose large language models (LLMs) generate accurate models of signaling networks. We find that general purpose LLMs generate 24–58% of the reactions of literature-curated networks for cardiomyocyte hypertrophy, myofibroblast activation, and mechano-signaling, and predicting network responses to perturbations with accuracies of 5–26%. While current general-purpose LLMs generate signaling networks with limited accuracy, this study provides a pipeline and benchmarks to guide future improvements.

The response of cells to external stimuli is a fundamental characteristic of life, mediated by complex molecular networks of signal transduction and gene regulation. Computational models of signaling networks have been widely used to understand their systems properties and new guide experiments^1,2^. However, a central hurdle is that computational models of signaling networks typically require substantial manual literature curation of network reactions and rate constants, which is time intensive and requires extensive experimental knowledge^3^. Further, existing data is often incomplete.

AI, and large language models (LLMs) in particular, have the potential to outperform human labor-intensive processes of knowledge curation. For example, general purpose LLMs have scored highly on the U.S. Medical Licensing Exam^4^, law exams^5^, and Advanced Placement exams for many subjects^5^. AI has also made substantial contributions in biology, including predicting protein structure^6,7^, cell types^8,9^, and is expected to play a central role in the development of comprehensive virtual cells^10–12^. However, to our knowledge, LLM-generated predictive models of signaling networks have not been reported or benchmarked. Here, we develop a pipeline for LLM-generated models of signaling networks, and we test whether general purpose LLMs generate networks with accurate structure and predictions of perturbation responses, as benchmarked against three manually-curated and validated models^13–15^.

We first established a computational pipeline to generate network models from LLMs using a given gene set (Figure 1A). For benchmarking purposes, here the gene set is based on annotations of manually curated network models that have been extensively validated. However, future studies could derive gene sets from other sources such as proteomics or transcriptomics. We optimized a programmatic LLM prompt that iteratively queried for directed (activation or inhibition) interactions among the gene products in the context of a particular phenotype (here “hypertrophy”, “fibroblast”, or “mechanosignaling”). With text processing, we extracted the returned directed interactions to examine network structure and then converted the network into the format needed to simulate network responses to perturbations, here with logic-based differential equations in Netflux^16^. LLM-based models were generated 10 times per network to examine reproducibility.

We first tested the extent to which three general purpose LLMs (GPT 4.1, Open AI; Gemini 2.0, Google; and Claude 3.7, Anthropic) could reconstruct the structure of the manually-curated cardiomyocyte hypertrophy signaling network^13^, based on its gene annotations alone. Overall, all three LLMs capture aspects of this manually-curated network, but with varying coverage depending on the region of the network and the particular LLM. In particular, GPT 4.1, Gemini 2.0, and Claude 3.7 all performed fairly well at reconstructing upstream aspects of ligand-receptor interactions (e.g. EGF to EGFR) and well-established second messenger cascades like β-adrenergic receptor to cAMP to CREB pathway or phospholipase C to diacylglycerol to PKC. They also accurately predicted the structure of highly conserved signaling axes such as PI3K-Akt-mTor and calcium-calmodulin-calcineurin-NFAT. In contrast, the three LLMs were less able to reconstruct regulation of downstream transcription factors (e.g. cFOS, NFAT, cJUN, MEF2) and gene expression (SERCA, αMHC, βMHC, natriuretic peptides ANP/BNP) or cell area / hypertrophy. The lower reconstruction accuracy of downstream signaling may arise because these contain more cardiomyocyte-specific reactions, but such gaps could also represent a general challenge of LLMs in reconstructing gene regulation from literature. Overall, the three LLMs exhibited 26.70–58.12% reconstruction of network reactions in the cardiomyocyte hypertrophy network. The top-performing LLM was Claude 3.7, which was reproducible with repeated queries.

To what extent is the limited recall of LLM-generated networks specific to cardiomyocyte hypertrophy? To address this question, we used GPT 4.1, Gemini 2.0, and Claude 4.7 to also reconstruct network models of fibroblast activation^14^ (91 nodes, 134 reactions) (Supplementary Figure 1) and mechanosignaling^15^ (94 nodes, 125 reactions) (Supplementary Figure S2). Like the hypertrophy model, these networks were previously manually reconstructed from the literature, translated into predictive logic-based models, and had their predictions validated against substantial experiments from the literature that were not used to develop the model. Consistent with the hypertrophy network, LLM-generated models of fibroblast activation and mechanosignaling successfully reconstructed 25.93–50.00% and 24.42–48.26% of their manually curated counterparts. Across all three networks, Claude 4.7 and GPT 4.1 outperformed Gemini 2.0, but with limited recall of the overall network.

We then asked whether the regional coverage of LLM-generated networks of fibroblasts and mechanosignaling mirrors that of cardiomyocytes. Indeed, the LLM-generated fibroblast network was most effective at reconstructing ligand-receptor interactions and highly conserved pathways such as cAMP and Ras/Raf/ERK, but could not recall AT1R signaling, PDGFR-abl signaling, and especially downstream regulation of transcription factors, genes, and extracellular matrix (e.g. MMP-collagen subnetwork) (Supplementary Figure 1). Unlike hypertrophy and fibroblast networks, mechanosignaling is mediated by stretch-dependent proteins that are relatively less characterized. Indeed, the LLM-generated networks for mechanosignaling did not adequately reconstruct direct mechano-sensation and had limited coverage of gene regulation consistent with the other networks (Supplementary Figure 2). Still, core conserved pathways for Na/Ca regulation, regulation of protein synthesis, and MAPK signaling were reconstructed well.

Models of signaling networks are useful not just for their description of structure, but especially for their ability to simulate the response to new perturbations. Making such predictions requires a specific mathematical formalism. For the three networks used for benchmarking, we had previously automatically translated them into logic-based differential equation models^17^ using the software Netflux^16^. This allows network-wide prediction of dynamics in response to any desired perturbations and does not require prior knowledge of rate constants or concentrations^17^. The predictions of these three logic-based models have also been highly validated against experiments not used to develop the model^13–15,18^. Therefore, we generated logic-based models based on all of the LLM-generated network structures described above.

Returning our focus to the hypertrophy network, we examined the ability of LLM-generated networks to predict classic the “fetal gene program” gene expression signature in cardiomyocytes. The manually curated model accurately predicts that AngII and/or ISO increase the expression of ANP, BNP, GATA4, βMHC, sACT, and increases cell area/size, while decreasing expression of SERCA and αMHC. In contrast, the LLM-generated network models produce limited aspects of this signature, with the best performance in predicting ISO-dependent ANP in the GPT version (Figure 2A). We then tested whether this validation accuracy scales across all available manually curated experiments that cover a wider array of perturbations and measured outputs. Overall, for 114 validations where the manually curated network has an functional accuracy of 94.74%, LLM-generated networks have an accuracy of 6.14–26.32% (Figure 2B). Repeating this systematic experimental validation for LLM-generated models of the fibroblast (Figure 2C) and mechano-signaling (Figure 2D), we observed similar validation accuracies of 16.87–21.69% and 5.81%, respectively. Together, these results indicate that validation accuracy of LLM-generated models for perturbation responses is even lower than their accuracy for reconstructing network structure. Because the validation tests are testing larger-range functional interactions, this difference may be caused by gaps in reconstructed pathways.

In conclusion, we developed a pipeline for generating predictive, logic-based models of signaling networks using general purpose large language models (LLMs). To test the reconstruction and simulation accuracy of LLM-generated network models, we benchmarked this pipeline using three LLMs (GPT 4.1, Claude 4.7, and Gemini 2.0) against three large-scale, manually curated and validated models of signaling networks. Across cardiac hypertrophy, fibroblast, and mechano-signaling networks, LLM-generated models exhibit 24.42–58.12% reconstruction of reactions from the manually curated models. Reconstruction accuracy is high for ligand-receptor interactions and well-conserved pathways, but lower for cell-specific gene regulation or biophysical regulation. Likely due to gaps in reconstructed pathways, LLM-generated models had a low ability to accurately simulate the responses of networks to perturbations. Overall, we provide a pipeline for LLM-generated models of signaling networks and benchmark their performance against three manually curated networks. Improving the performance of LLM-generated network models may require hybrid manual-LLM approaches or advances in more special-purpose AI models.

## Methods

### Prior knowledge models of signaling networks

To benchmark LLM-generated network models, we selected three large-scale curated network models based on manual curation of the directed interactions using the literature^13–15^. These models were recently used to benchmark protein interaction databases^19^, creating a comparison` for the current analysis. These models contain a range of biological processes including ligand-receptor interactions, phosphorylation, gene regulation, and cellular phenotypes. Associated with the network structure is an annotated list of genes associated with the nodes in the model. These models were mathematically formulated as systems of logic-based differential equations^17^, which enable dynamic network-wide predictions of how those cells respond to new perturbations. The predictive accuracy of these models has previously been rigorously tested with manual curation of literature not used to develop the models, with accuracies of 94.74% (hypertrophy), 81.93% (fibroblast), and 77.33% (mechanosignaling)^13–15^.

### Querying large language models

We focused on widely used, general-purpose large language models (LLMs) that are most relevant to wider use in the field. Specifically, we selected current versions of GPT (4.1, OpenAI), Gemini (2.0, Google), and Claude (3.7, Anthromorphic). The prompt consisted of three steps:

1. The list of gene symbols, along with the description: “List of genes and other signaling nodes:”.
2. Description of the desired outputs: “For the first {batch_size} entries in this list of genes, proteins, and other signaling nodes from a {phenotype} network, please provide more than 0 but fewer than {max_connections} direct interactions with other nodes from the list supported by available literature. Simply list the input node, affected node, and if the affected node is stimulated / inhibited.” Batch size was set at 20 genes, max connections set to the maximum number of connections exhibited in that parent network, and phenotype set at “cardiac hypertrophy”, “fibroblast”, or “mechanosignaling” corresponding to the three networks used for benchmarking.
3. Instructions to repeat the process until reaching the end of the gene list: “That looks great! Please do the same operation for the next {len(chunk)} nodes! Thank you”
4. The responses were stored as .txt files and then programmatically extracted using regular expressions.

### Extracting reactions from LLM outputs, logic-based model generation

Each of the LLMs returned network reactions in slightly different format. But for example, Claude 3.7 returned reactions in the form: “ADRB2 stimulates RAC1” or “RAC1 stimulates MAP3K1”. Relationships based on directional signed relationships were kept, such as terms “stimulates”, down-regulates”, or “generates”. Returned reactions referring to non-relevant proteins or processes were discarded, as were non-directional relationships. We attempted also querying PubMed IDs, but these were rarely accurate.

Reactions successfully extracted from the LLM outputs were formatted for uniform comparison to the reaction list from the corresponding manually curated network. Finally, this reaction list was translated into the Netflux format for automated conversion to a logic-based differential equation model^16,17^. This final logic-based model was used to perform validation simulations.

## Supplementary Material

Supplement 1

## Figures and Tables

**Figure 1:**
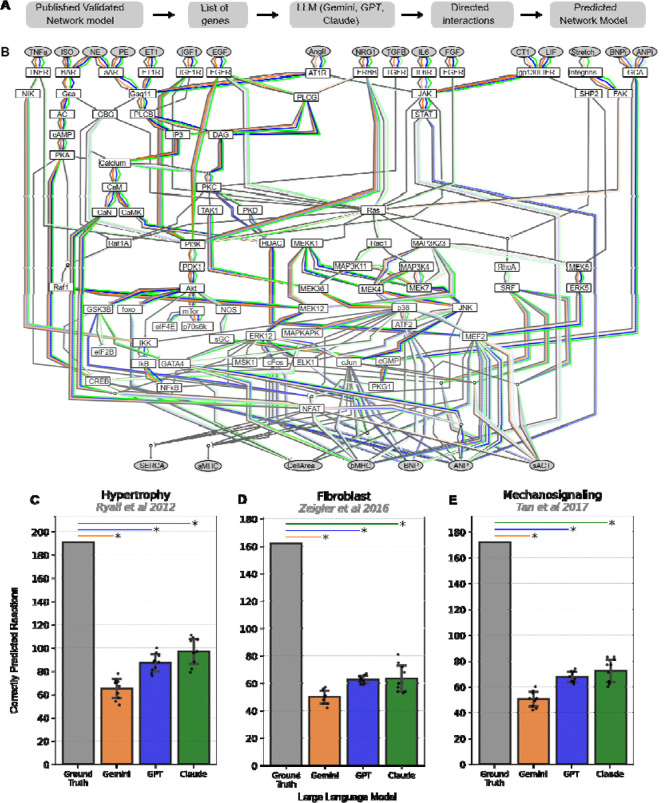
Signaling networks generated by general-purpose large language models. A) Schematic of pipeline for LLM-generated models of signaling networks. B) Network reactions recalled by three large language models (Gemini2.0, orange; ChatGPT4, blue; Claude3.7, green) compared with a “Ground Truth” literature-curated and validated cardiomyocyte hypertrophy signaling network^13^ (gray reactions). LLM networks were generated using iterative prompts based on the gene set list of the Ground Truth hypertrophy network. Summary of reaction recall accuracy for three literature-curated signaling networks (C, hypertrophy^13^; D, fibroblast^14^; and E, mechanosignaling^15^) by Gemini, GPT, and Claude. * Indicates p < 10^−9^ in one-sample T test between LLM-generated replicates (n = 10) and the ground truth network.

**Figure 2:**
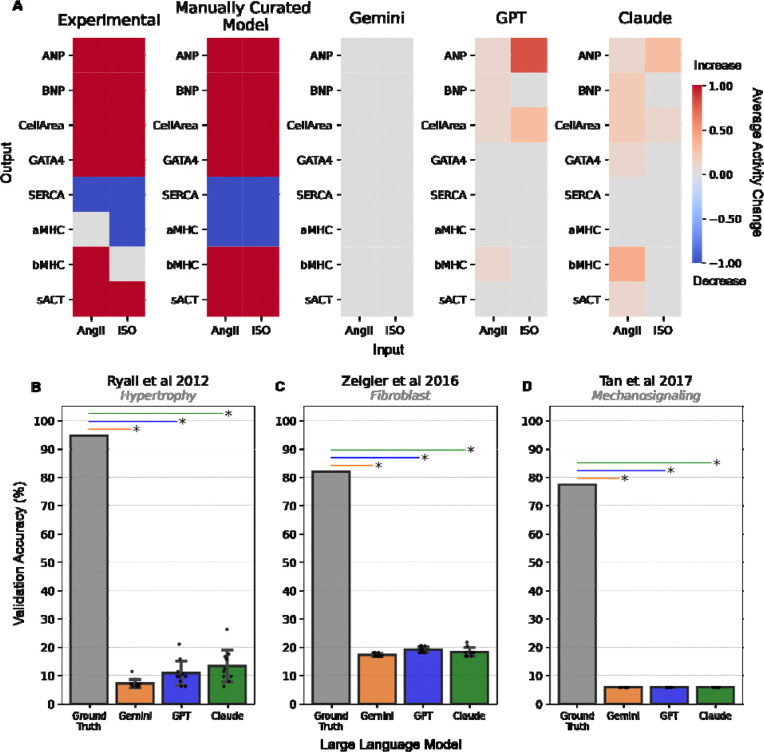
Experimental validation of perturbation responses predicted by LLM-generated signaling network models. A) Representative validations of network models generated by manual curation or by LLMs (Gemini, GPT, Claude), in comparison to experiments in conditions of Angiotensin II (AngII) or isoproterenol (ISO) from the literature^13^. B) Summary of systematic validations of manually curated (Ground Truth) and LLM-generated network models of hypertrophy, fibroblast, and mechano-signaling network models against perturbation experiments from the literature (n = 114, 83, and 171 experiments, respectively). * Indicates p < 10^−11^ in one-sample T test between LLM-generated model validation scores (n = 10 replicates) and ground truth model validation accuracy.
