# Supplementary material for: Benchmarking of signaling networks generated by large language models
Source: bioRxiv. 2025 Jul 29:2025.07.28.667217. Preprint. [Version 1] doi: 10.1101/2025.07.28.667217 (PMC12324320; doi:10.1101/2025.07.28.667217)
Supplement: Supplement 1 [file NIHPP2025.07.28.667217v1-supplement-1.pdf]

bioRxiv preprint doi: https://doi.org/10.1101/2025.07.28.667217; this version posted July 29, 2025. The copyright holder for this preprint (which was not certified by peer review) is the author/funder, who has granted bioRxiv a license to display the preprint in perpetuity. It is made available under aCC-BY 4.0 International license.

Supplementary Information

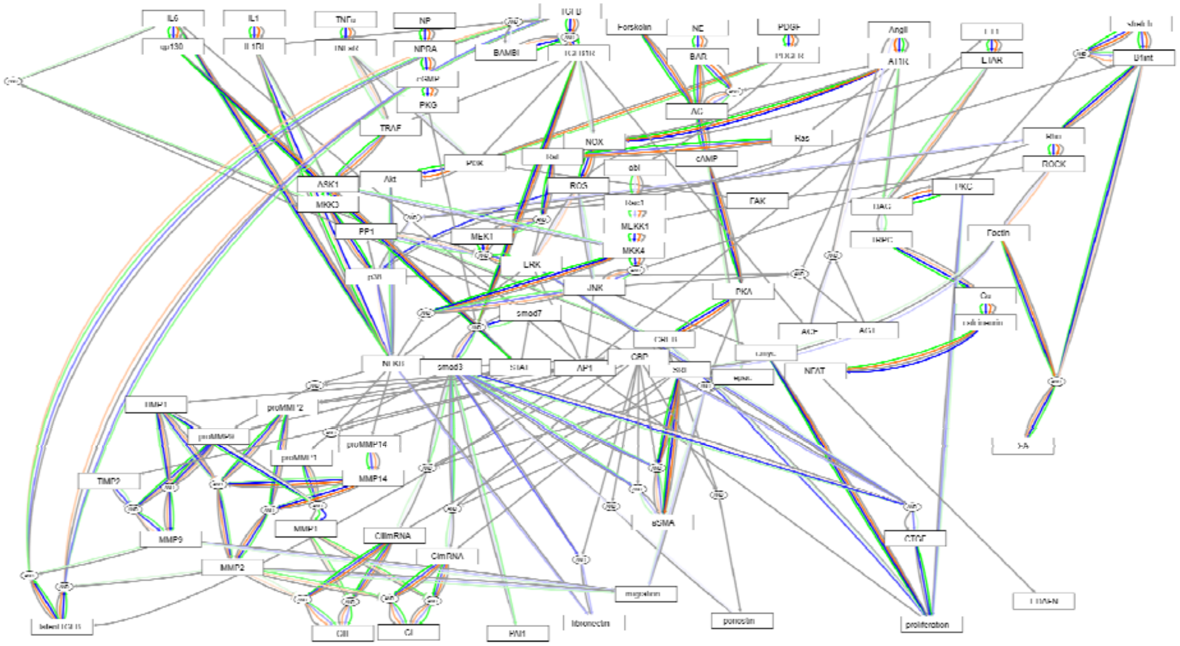

Supplementary Figure 1. Visualization of LLM-generated fibroblast signaling networks, as recalled by three general-purpose large language models. Network reactions recalled by three large language models (Gemini2.0, orange; ChatGPT4, blue; Claude3.7, green) compared with a “Ground Truth” literature-curated and validated fibroblast signaling network (gray reactions). LLM-generated networks used prompts based on the gene set list of the Ground Truth fibroblast network. This visualization corresponds to the analyses in **Figure 1D**.

bioRxiv preprint doi: https://doi.org/10.1101/2025.07.28.667217; this version posted July 29, 2025. The copyright holder for this preprint (which was not certified by peer review) is the author/funder, who has granted bioRxiv a license to display the preprint in perpetuity. It is made available under aCC-BY 4.0 International license.

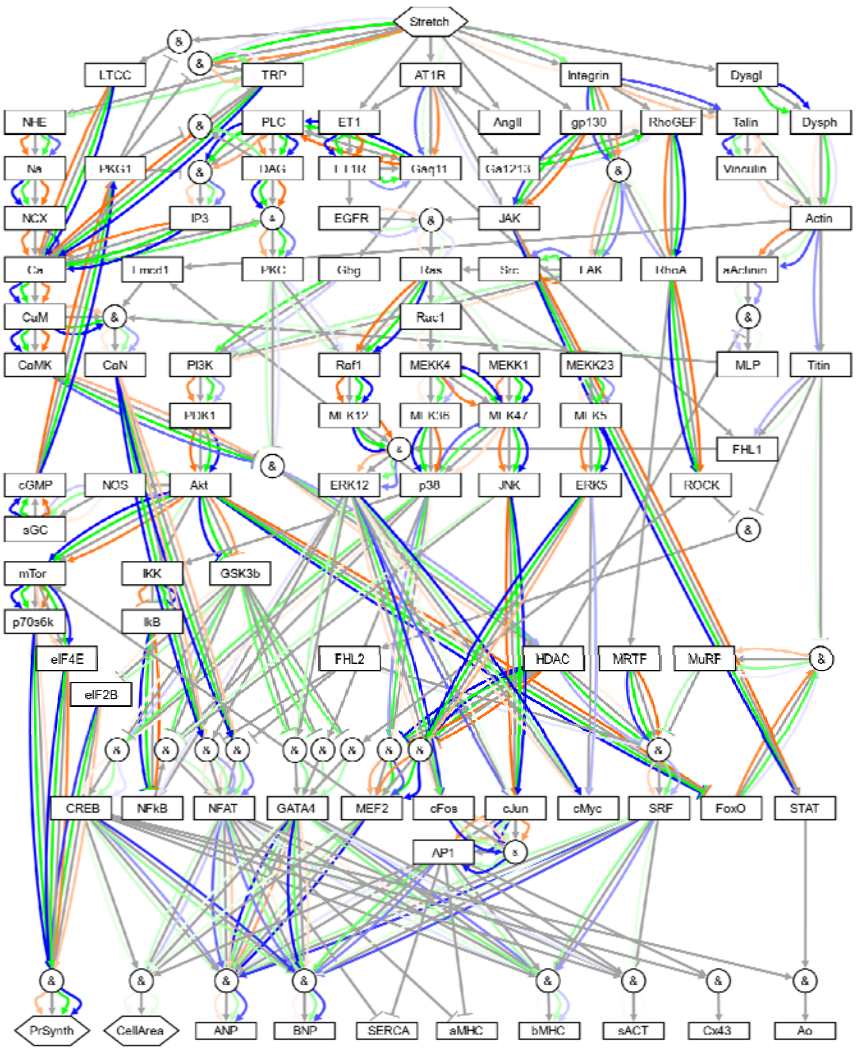

Supplementary Figure 2. Visualization of LLM-generated mechanosignaling networks, as recalled by three general-purpose large language models. Network reactions recalled by three large language models (Gemini2.0, orange; ChatGPT4, blue; Claude3.7, green) compared with a “Ground Truth” literature-curated and validated mechanosignaling network (gray reactions). LLM-generated networks used prompts based on the gene set list of the Ground Truth mechanosignaling network. This visualization corresponds to the analyses in **Figure 1E**.
